# Effects of Virgin Olive Oil on Bone Health in Ovariectomized Rats

**DOI:** 10.3390/nu12051270

**Published:** 2020-04-30

**Authors:** Manuel Díaz-Curiel, Blanca Torrubia, Marta Martín-Fernández, Mercedes Rubert, Concepción De la Piedra

**Affiliations:** 1 Internal Medicine, Instituto de Investigación Sanitaria Fundación Jiménez Díaz, 28040 Madrid, Spain; mdcuriel@fjd.es; 2 Biochemistry Research, Instituto de Investigación Sanitaria Fundación Jiménez Díaz, 28040 Madrid, Spain; blanca.torrubia@quironsalud.es (B.T.); martamartinfernandez@gmail.com (M.M.-F.); 3 Hospital Support Team, Palliative Care, Hospital Universitario de Móstoles, 28935 Móstoles, Madrid, Spain; merche.rubert@gmail.com

**Keywords:** osteoporosis, olive oil, fractal dimension, Young’s modulus

## Abstract

Osteoporosis is a pressing concern facing public health, thus making research into the effects of nutrients on bone health particularly important. Evidence from preclinical studies using animal models and a limited number of studies in human suggests that olive oil (OO) is a protective agent for bone. The aim of this work is to study the effects of virgin olive oil (VOO) consumption by ovariectomized rats on bone health. A total of 48 6-month-old female Wistar rats weighing 320 ± 10 g (mean ± SD) were divided into the following groups: SHAM (n = 12), simulated intervention; OVX (n = 12), ovariectomized; OVX + 100 (n = 12), ovariectomized and treated with VOO (100 µL/day by oral gavage); and OVX + 200 (n = 12) ovariectomized and treated with VOO (200 µL/day by oral gavage), all over 3 months. Femoral (F) and lumbar (L) bone mineral density (FBMD and LBMD), microtomographic parameters, fractal dimension D2D and D3D, and biomechanical properties were studied. After 3 months of VOO treatment, although FBMD and LBMD were not affected, bone quality was improved, as the elasticity of bone and fractal dimension (complexity of bone) were more similar to healthy bone. Our results support the findings of previous research suggesting that dietary intake of olive oil may exert beneficial effect on some bone characteristics.

## 1. Introduction

Osteoporosis is one of the major problems facing public health systems [1], and it is therefore essential to take all necessary actions to mitigate the development of this disease. Bone modeling and remodeling are governed by nutritional and hormonal status [2], among other factors. Nutrition exerts various relevant effects on peak bone mass, age-related bone loss, and muscle strength, to name a few (1). The primary nutrients influencing bone modeling and remodeling are calcium and vitamin D [3]. However, the European Union has acknowledged the relevance of other nutrients and has called for further research on their effectiveness in bone [4]. Nutrition is of particular importance because it is modifiable.

Several studies have found that the Mediterranean diet (MD), which is characterized by a high intake of fruits, vegetables, and olive oil (OO), is associated with better bone health [5]. OO is the main dietary staple among the Mediterranean population, where it accounts for one third the total intake of vegetable fats [6].

The incidence of osteoporosis and associated fractures has been found to be lower in countries where the MD is predominant [5]. The most widely held view is that this rate may be linked to the effect of the active components of virgin olive oil (VOO). Adherence to the MD has been positively related to bone mass, suggesting the potential bone-preserving properties of this pattern through adult life [7]. Adherence to the MD had a beneficial effect on bone mineral density (BMD) in the calcaneus in a sample of healthy women in southern Spain [8]. In addition, Savanelli et al. studied 418 healthy people (105 males and 313 females) aged 50 ± 14 years, demonstrating a positive correlation between bone health status and MD adherence [9]. The problem with studies such as the two mentioned above is that they are designed in reference to the MD, though an assumption of our study is that VOO is the responsible component; however, fruits and vegetables are also important components of this diet, and as a result their effects cannot be disregarded. Silva et al. studied 105 healthy postmenopausal women aged 45 to 65 years [10]. Women with higher adherence to the MD had a higher lumbar spine BMD, *p* = 0.007.

We performed a literature search for all relevant publications since 2001 in MEDLINE, EMBASE, and the Cochrane Library databases using the descriptors Mediterranean diet, VOO, phenols, bone, osteoblast, and osteoporosis. The results of this search suggest that OO phenols confer benefits by preventing loss of bone mass. It has been demonstrated that these phenols can modulate the proliferative capacity and cell maturation of osteoblasts by increasing alkaline phosphatase activity and depositing calcium ions in the extracellular matrix [11].

In addition to triglycerides, VOO contains a wide variety of so-called minor components, including phenolic compounds. Oleuropein is the main phenolic compound in OO [12]. One of the most important groups of bioactive metabolites in VOO are phenolic alcohols such as tyrosol and hydrotyrosol [13]. Flavonoids, including lutein, are also abundant in VOO [14].

Olives, OO, or olive polyphenols can act as bone protective agents. This is supported by evidence derived from preclinical studies using animal models of osteoporosis and a limited number of human studies [15].

The aim of this work is to study VOO intake in ovariectomized rats, examining its effects on bone quality as measured by BMD, microtomographic parameters, fractal dimension D2D and D3D, and biomechanical parameters.

## 2. Material and Methods

### 2.1. Animals

A total of 48 6-month-old female Wistar rats weighing 320 ± 10 g (mean ± SD) were used in this study. The animals were kept under constant living conditions (22 °C, 12-h light-dark cycles per day), and food (standard laboratory chow) and water were available *ad libitum*.

The animals were randomized into the following groups: SHAM (n = 12), simulated intervention; OVX (n = 12), ovariectomized; OVX + 100 (n = 12), ovariectomized and treated with OO (100 µL/day by oral gavage); and OVX + 200 (n = 12) ovariectomized and treated with OO (200 µL/day by oral gavage), all over 3 months. The dosages were chosen thinking in a quantity of OO that could be ingested in a normal Mediterranean diet by a person of 60 kg. A total of 100 µL/ or 200 µL/day in a rat of 320 g is equivalent to 6 or 12 mL in a person of 60 kg. Commercial VOO was used, (Aceite de Oliva Virgen Los Cerros de Úbeda, Cooperativa La Carrera, Úbeda, Jaen, Spain). Treatment began the first day following the ovariectomy. The rat chow, SAFE A04 (Augy, France), contained 7.3 g/kg of calcium and 1000 IU/kg of vitamin D3. No animals died during the study. One day after the last treatment, the experimental animals were weighed and killed by exsanguination under isoflurane (Florane^®^) anesthesia. Blood samples were obtained by cardiac puncture and serum samples were immediately frozen at –80 °C as aliquots until biochemical markers of bone turnover were measured. Once the blood was collected, the animals were frozen at –20 °C until determination of BMD in previously thawed animals. Prior to BMD analyses, the left femurs were excised and cleaned of adjacent tissue for BMD determination. Right femurs were also excised and cleaned of adjacent tissue and used for computerized microtomography and biomechanical testing. Lumbar spine BMD was determined in situ. It has been shown that the use of repeated freeze-thaw cycles does not influence the structural properties of bone [16]. All procedures were carried out in accordance with European Community standards for the care and use of laboratory animals and after approval of the ethics committee of the Health Research Institute-Fundación Jiménez Díaz, according to the Spanish legislation relative to use, care and protection of experimental animals (Royal Decree-law 1201/2005).

### 2.2. Bone Mineral Density

BMD was determined in situ in the lumbar spine (L2, L3, and L4) and in the entire right femur by dual-energy x-ray densitometry (DEXA) using a Piximus densitometer (HOLOGIC QDR-1000 TM) with small-animal software. Intra- and inter-assay coefficients of variation (CV) were <0.53% and < 1.2%, respectively. The scans of the femur were analyzed to determine the BMD of the whole femur. Scans of the L2, L3, and L4 vertebrae were analyzed for BMD, and the results were expressed as mean values.

### 2.3. Trabecular Microarchitecture Analysis of Femur

Trabecular bone microarchitecture was performed using the QUIBIM SL system (Quantitative Imaging Biomarkers in Medicine, Valencia, Spain) with computerized microtomography.

We used a GE system for in vitro samples (eXplore Locus SP^®^, General Electric, USA), which includes technology for plane detection by means of cone-beam (CT). The spatial resolution of the acquired images was isotropic, with voxels of 50 × 50 × 50 µm. Although the voxel had a limited spatial resolution of 50 µm, this was considered adequate for the study of trabecular thickness assuming that the measurement would take into account partial volume effect and given that previous studies have used larger voxels (290 µm) to measure human trabecular thickness (80–150 µm), obtaining significant correlation with mechanical properties [17]. The primary spongiosa was excluded from the analysis. Segmentation of the µCT samples was performed by placing a rectangular region of interest and automatically propagating this position to the rest of the slices, verifying that all regions were segmented in each femur, that is, femoral head, trochanter, and distal femoral metaphysis (near the knee). Otsu’s method was used for thresholding intensities [18].

The following morphometric parameters of trabecular bone were determined: bone volume/total bone volume (BV/TV), trabecular thickness (Tb.Th), trabecular number (Tb.N), and trabecular separation (Tb.Sp).

We also calculated irregularity through fractal dimension analysis in 2D (D2D) and in 3D (D3D), expressing the degree of complexity of the contour of a structure in the fill of a surface or a volume, respectively [19].

### 2.4. Biomechanical Analysis

Samples of trabecular bone reconstructed from µCT images were subjected to simulation of mechanical compression by finite-element analysis. Static simulations of uni-axial compression in the 3 axes were performed on the different virtual samples obtained by tension-deformation assay. Properties of the basic material were defined from those of compact bone (Young’s modulus E = 10 GPa; Poisson’ ratio, σ = 0.3). In the simulations of compression assays, we applied null displacement on nodes from one side, and a displacement of 1% of the edge length of the opposite side. Using the resolution of the equation systems, the apparent elastic modulus of the structure (Young’s modulus) was obtained in three directions (Ex, Ey, Ez) [20].

### 2.5. Biochemical Markers of Bone Turnover

Serum bone gla-protein (BGP) was measured by ELISA for specific quantitative determination of rat osteocalcin levels (Rat-MID Osteocalcin, IDS, UK). The sensitivity of this assay was 50 ng/mL, and intra- and inter-assay coefficients of variation were < 5.0% and < 6.6%, respectively.

Serum 5b isoenzyme of tartrate-resistant acid phosphatase (TRAP) was measured by a specific rat TRAP ELISA kit (RatTRAP Assay, IDS, UK). The sensitivity of the assay was 0.1 U/L. The intra- and inter-assay coefficients of variation for the method were < 5.0% and < 5.5%, respectively.

### 2.6. Weight of the Rats

All rats were weighed at the beginning and the end of the experiment.

### 2.7. Statistical Analysis

Sample size calculation was made using OpenEpi V2 open source calculator. For a two-sided 95% Confidence interval and an 80% power, a total of 12 animals per group was required.

Initially, the conditions of applicability of variance analysis (ANOVA) were studied. That study was realized by the test of Kolmogorov Smirnov. We choose that analysis because the number of each group was 12, lower than 30.

After this study, and confirming that the different groups did not have a normal distribution, the study of homogeneity of variances was not necessary and all the study was realized with nonparametric tests.

Initially, a global comparison of all the groups was done for each parameter, to reject the groups proceeding of populations with the same mean value for all the parameters. We used the global test of Kruskal Wallis.

Finally, groups were compared between them by the nonparametric test of Mann-Whitney, penalized by the method of Bonferroni to avoid the addition of errors. The cut point to stablish significant differences was *p* < 0.05.

We expressed the results in graphics, representing mean values ± standard deviation of each parameter.

We applied the programme “Estadística Aplicada en Ciencias de la Salud”, Instituto IMDEA, (Universidad Autónoma , Madrid)

## 3. Results

### 3.1. Effect of VOO on FBMD and LBMD

**Figure 1:**
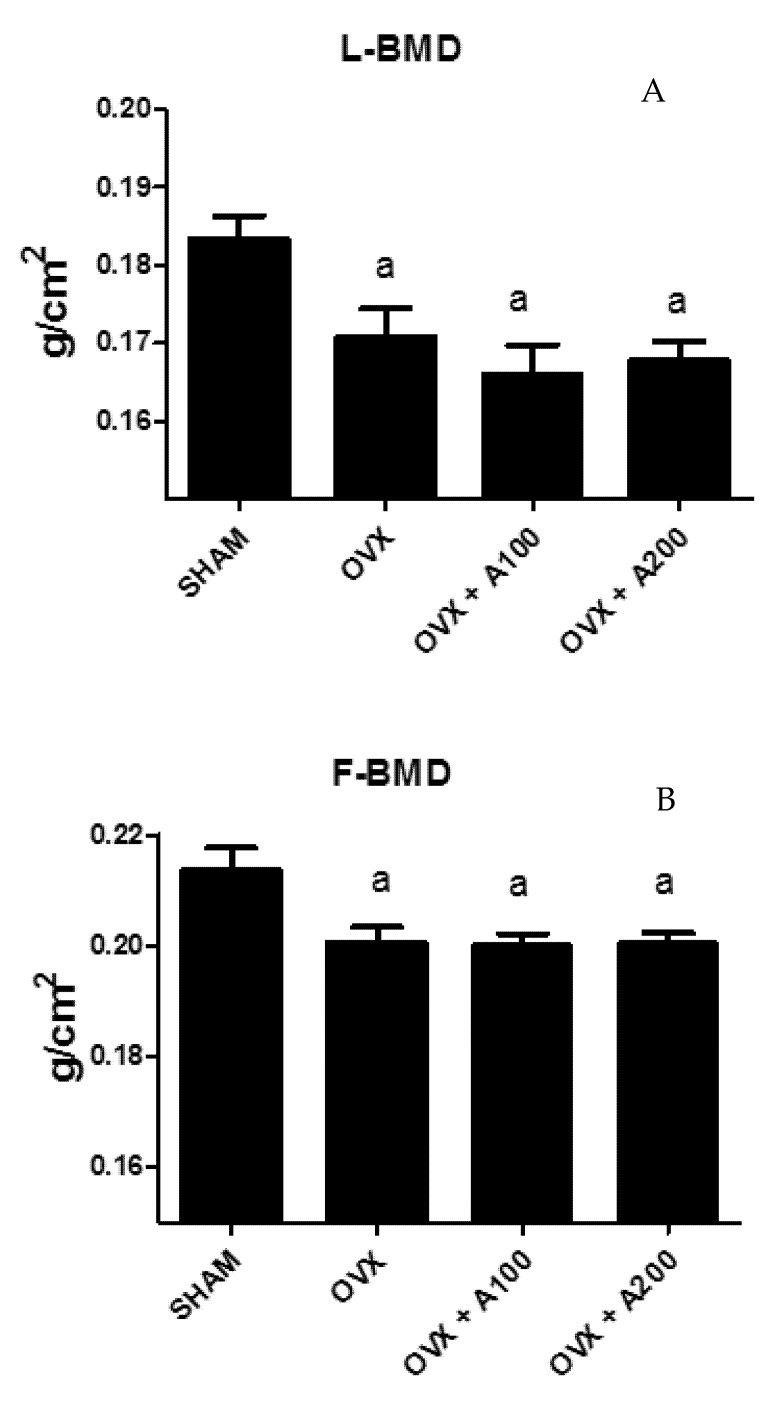
Lumbar bone mineral density (LBMD) (**A**) and femoral BMD (FBMD) (**B**) in a group of SHAM operated, ovariectomized (OVX), ovariectomized and treated with olive oil (OVX + 100) (100 µL/day by oral gavage), and ovariectomized and treated with olive oil (OVX + 200) (200 µL/day by oral gavage) over 3 months, n = 12. Data are expressed as mean ± SD. Statistical significance: (**a**) vs. SHAM. FBMD: OVX vs. SHAM *p* < 0.05, OVX + 100 vs. SHAM *p* < 0.05, OVX + 200 vs. SHAM *p* < 0.05. LBMD: OVX vs. SHAM *p* < 0.05, OVX + 100 vs. SHAM *p* < 0.01, OVX + 200 vs. SHAM *p* < 0.01.

Figure 1 shows the values of FBMD in the groups of rats studied. As expected, both F and LBMD decreased significantly in ovariectomized rats. This decrease was not recovered with treatment with VOO. LBMD was lower in OVX than in SHAM rats (0.170 ± 0.03 vs. 0.185 ± 0.02, *p* < 0.05) and LMBD of rats treated with olive oil was also lower than that of SHAM rats (0.168 ± 0.03, 0.169 ± 0.02 vs. 0.185 ± 0.02, *p* < 0.01) without differences with OVX rats. FBMD was lower in OVX and OVX treated with oil rats (0.200 ± 0.03, 0.200 ± 0.03, 0.200 ± 0.02 vs. 0.217 ± 0.04, *p* < 0.05) without significant differences between them.

### 3.2. Effect of VOO on Microtomographic Parameters

**Figure 2:**
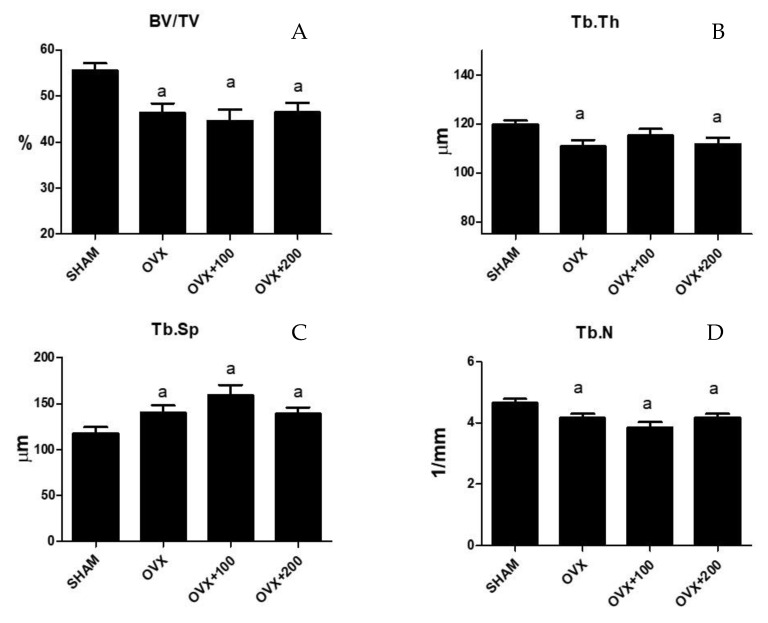
Femoral trabecular microarchitecture analysis: bone volume/total volume (BV/TV) (**A**), trabecular thickness (Tb.Th) (**B**), trabecular separation (Tb.Sp) (**C**), and trabecular number (Tb.N) (**D**). SHAM operated, ovariectomized (OVX), ovariectomized and treated with olive oil (OVX + 100) (100 µL/day by oral gavage) and ovariectomized and treated with olive oil (OVX + 200) (200 µL/day by oral gavage) over 3 months, n = 12. Data are expressed as mean ± SD. Statistical significance: (**a**) vs. SHAM. BV/TV: OVX vs. SHAM *p* < 0.01, OVX + 100 vs. SHAM *p* < 0.001, OVX + 200 vs. SHAM *p* < 0.001. Tb.Th: OVX vs. SHAM *p* < 0.01, OVX + 200 vs. SHAM *p* < 0.05. TbSp: OVX vs. SHAM *p* < 0.05, OVX + 100 vs. SHAM *p* < 0.01, OVX + 200 vs. SHAM *p* < 0.05. TbN: OVX vs. SHAM *p* < 0.05, OVX + 100 vs. SHAM *p* < 0.01, OVX + 200 vs. SHAM *p* < 0.05.

Figure 2 shows the results of microtomographic analysis. Bone volume/total volume (BV/TV), trabecular thickness (Tb.Th), and trabecular number (TbN) decreased (53.99 ± 7.72 vs. 63.28 ± 1.13 *p* < 0.01, 110.14 ± 11.48 vs. 120.80 ± 4.17, *p* < 0.01, and 4 ± 0.29 vs. 4.5 ± 0.27, *p* < 0.05 respectively), and trabecular separation (Tb.Sp) increased significantly (140.8 ± 31.8 vs. 110 ± 4, *p* < 0.05) in ovariectomized rats. The situation was similar in the groups of rats treated with 100 µL or 200 µL of VOO, with the exception of Tb.Th in the group treated with 100 µL of VOO, which showed a similar value to the SHAM group (BV/TV in OVX + 100:58 ± 2.5; BV7TV in OVX + 200 57 ± 1). (Tb.Th in OVX + 100: 119 ± 12.4; Tb.Th in OVX + 200:111 ± 9).(Tb.Sp in OVX + 100:150 ± 11; Tb.Sp in OVX + 200: 140 ± 8) (TbN in OVX + 100:3.9 ± 0.4; Tb.N in OVX + 200: 4 ± 0.3).

### 3.3. Effect of VOO on Fractal Dimension

**Figure 3:**
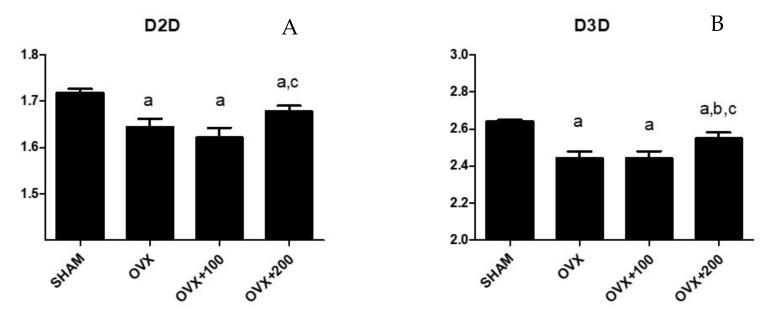
Fractal dimension analysis in 2D (D2D) (**A**) and in 3D (D3D) (**B**). SHAM operated, ovariectomized (OVX), ovariectomized and treated with olive oil (OVX + 100) (100 µL/day by oral gavage) and ovariectomized and treated with olive oil (OVX + 200) (200 µL/day by oral gavage) over 3 months, n = 12. Data are expressed as mean ± SD. Statistical significance: (**a**) vs. SHAM; (**b**) vs. OVX, (**c**) vs. OVX + 100. D2D: OVX vs. SHAM *p* < 0.01, OVX + 100 vs. SHAM *p* < 0.01, OVX + 200 vs. SHAM *p* < 0.05, OVX + 200 vs. OVX + 100 *p* < 0.05. D3D: OVX vs. SHAM *p* < 0.01, OVX + 100 vs. SHAM *p* < 0.01, OVX + 200 vs. SHAM *p* < 0.05, OVX + 200 vs. OVX *p* < 0.05, OVX + 200 vs. OVX + 100 *p* < 0.05.

Figure 3 shows the values of D2D and D3D for the groups SHAM, OVX rats, and rats treated with 100 or 200 µL of VOO. The group of rats treated with 200 µL of VOO presented a higher value of D2D than that of OVX + 100 rats (*p* < 0.05) (1.69 ± 0.07 vs. 1.64 ± 0.02), although this D2D was lower than the SHAM group (1.72 ± 0.02) (*p* < 0.05). This same group of rats had a higher D3D value than the groups of OVX *p* < 0.05) and OVX + 100 rats (*p* < 0.05) though lower (*p* < 0.05) than the SHAM group. (D3 SHAM: 2.67 ± 0.03; D3 OVX: 2.4 ± 0.11; D3 OVX + 100: 2.4 ± 0.02; D3 OVX + 200: 2.55 ± 0.15).

### 3.4. Effect of VOO on Young’s Modulus

**Figure 4:**
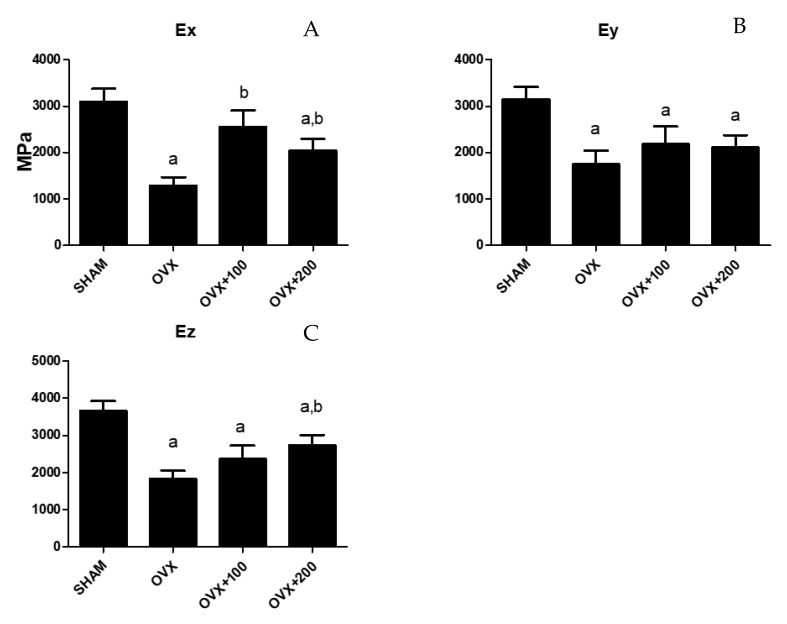
Young´s modulus of the femur obtained in three directions (Ex (**A**), Ey (**B**), Ez (**C**)). SHAM operated, ovariectomized (OVX), ovariectomized and treated with olive oil (OVX + 100) (100 µL/day by oral gavage) and ovariectomized and treated with olive oil (OVX + 200) (200 µL/day by oral gavage) over 3 months, n = 12. Data are expressed as mean ± SD. Statistical significance: (**a**) vs. SHAM; (**b**) vs. OVX. Ex: OVX vs. SHAM *p* < 0.001, OVX + 100 vs. OVX *p* < 0.05, OVX + 200 vs. SHAM *p* < 0.05, OVX + 200 vs. OVX *p* < 0.05. Ey: OVX vs. SHAM *p* < 0.01, OVX + 100 vs. SHAM *p* < 0.01, OVX + 200 vs. SHAM *p* < 0.01. Ez: OVX vs. SHAM *p* < 0.001, OVX + 100 vs. SHAM *p* < 0.01, OVX + 200 vs. SHAM *p* < 0.05, OVX + 200 vs. OVX *p* < 0.05.

Figure 4 shows the values for Ex, Ey, and Ez in the four groups of rats studied. Young’s modulus decreased in the directions x, y, and z in OVX rats (Ex: 1000 ± 150 vs. 3000 ± 200, *p* < 0.001.; Ey: 1600 ± 300 vs. 3000 ± 200, *p* < 0.01; Ez:1900 ± 50 vs. 3800 ± 100, *p* < 0.001 respectively). Treatment with 100 µL of VOO caused the value to recover in the x-axis (2600 ± 300), and treatment with 200 µL of VOO produced a Young’s modulus that was higher than that of OVX groups in the z-axis (2500 ± 100, *p* < 0.05) although lower (*p* < 0.05) than that of the SHAM group.

### 3.5. Effect of VOO on Biochemical Markers of Bone Turnover

**Figure 5:**
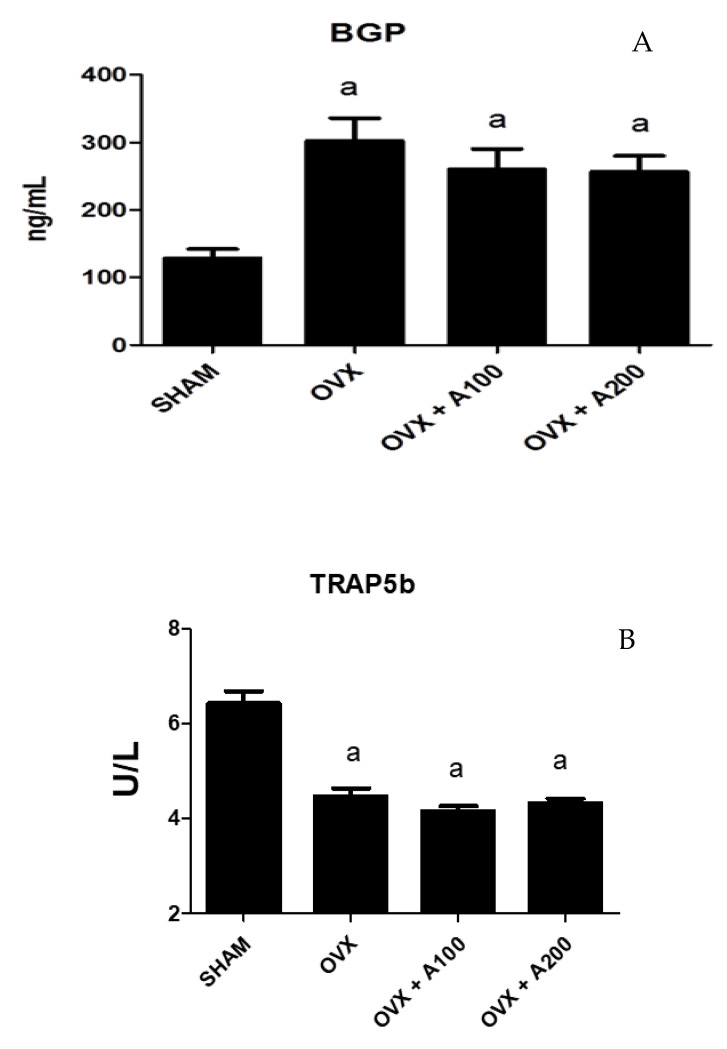
Serum osteocalcin (BGP) (**A**) and isoenzyme b of serum tartrate-resistant acid phosphatase (TRAP) (**B**). SHAM operated, ovariectomized (OVX), ovariectomized and treated with olive oil (OVX + 100) (100 µL/day by oral gavage) and ovariectomized and treated with olive oil (OVX + 200) (200 µL/day by oral gavage) over 3 months, n = 12. Data are expressed as mean ± SD. Statistical significance: (a) vs. SHAM; BGP: OVX vs. SHAM *p* < 0.001, OVX + 100 vs. SHAM *p* < 0.01, OVX + 200 vs. SHAM *p* < 0.001. TRAP: OVX vs. SHAM *p* < 0.001, OVX + 100 vs. SHAM *p* < 0.001, OVX + 200 vs. SHAM *p* < 0.001.

Figure 5 shows the biochemical markers of bone turnover in the groups of rats studied. BGP increased significantly in the OVX group (300 ± 15 vs. 120 ± 17, *p* < 0.001), showing no differences after treatment with VOO. TRAP5b decreased significantly in the OVX group (4.8 ± 0.8 vs. 6.5 ± 1, *p* < 0.001), with no differences observed with VOO treatment.

### 3.6. Effect of VOO on Final Weight of the Rats

**Figure 6:**
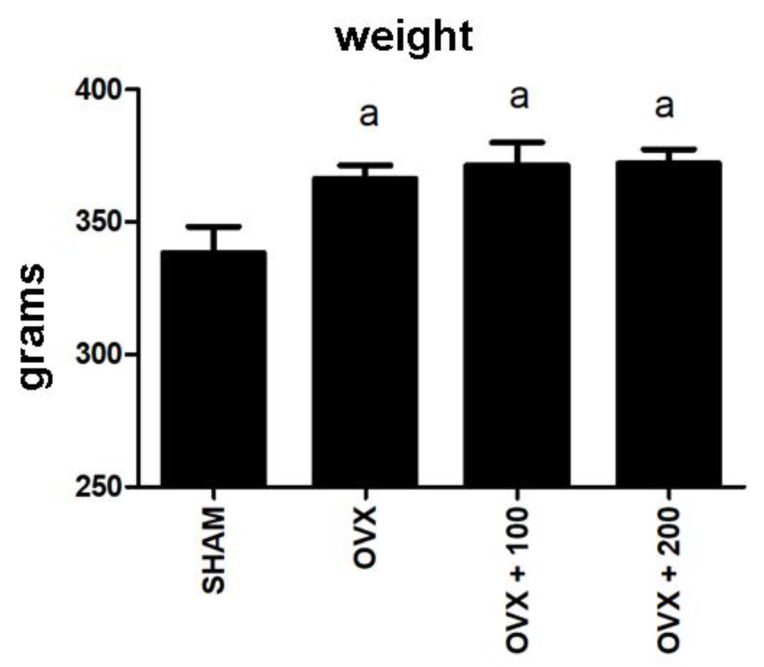
Weights at the end of the experiment. SHAM operated, ovariectomized (OVX), ovariectomized and treated with olive oil (OVX + 100) (100 µL/day by oral gavage) and ovariectomized and treated with olive oil (OVX + 200) (200 µL/day by oral gavage) over 3 months, n = 12. Data are expressed as mean ± SD. Statistical significance: (**a**) vs. SHAM; OVX vs. SHAM *p* < 0.05, OVX + 100 vs. SHAM *p* < 0.05, OVX + 200 vs. SHAM *p* < 0.01.

Figure 6 shows the weight of the rats at the conclusion of the experiment. As expected, ovariectomy produced a significant increase in the weight of the rats (*p* < 0.05), and OO intake did not revert this change.

## 4. Discussion

Centering our study on olive oil consumption, Roncero et al. examined 523 women ranging in age from 23 to 81 years, dividing the sample according to VOO intake above or below 18.32 g/day [21]. The authors found significant increases in volumetric BMD (*p* < 0.001) in the group with a higher intake of olive oil. In another study in adult females in Spain, Rivas [22] found a significant correlation between the consumption of monosaturated fatty acids derived from OO and the mineral density of the heel bone (calcaneous).

We will now discuss some existing studies with animals investigating the effects of OO on bone. Ostrowska et al. observed that pigs fed with VOO (19% w/w) experienced a daily increase of 6.28 mg/cm^2^ in BMD [23]. For their part, Saleh and Saleh studied female Wistar rats aged 12 to 14 months [24]. These rats received an oral VOO supplement for 12 weeks (1 mL/100 g body weight) 4 weeks before undergoing ovariectomy and 8 weeks after the procedure. OVX animals displayed a significant decrease in cortical and trabecular thickness and a significant increase in osteoclast number. These parameters were markedly improved in the group treated with VOO.

Oleuropein has been found to enhance bone health by increasing the formation of osteoblasts from bone marrow stem cells [25]. Puel et al. evaluated the effect of oleuropein in a model of OVX rats with and without inflammation [26]. This phenolic compound (0.15 g oleuropein/kg/day) was able to elicit protective effects on bone loss only in the model with inflammation.

Hydroxytyrosol administered at a dose of 50 µM to 100 µM inhibits the formation of multinucleated osteoclasts in a dose-dependent manner [27].

Luteolin, a flavonoid present in VOO decrease the differentiation of bone marrow nuclear cells into osteoclasts [14]. Melguizo-Rodriguez et al. demonstrated that luteolin, apigenin, p-coumaric, caffeic or ferulic acid (phenolic compounds present in olive oil) added to a MG-63 osteoblast cell line induced cell maturation in vitro, increased alkaline phosphatase synthesis, and reduced the expression of antigens involved in the immune functions of osteoclasts [28].

In the present work, we did not observe any improvement in LBMD or FBMD among the OVX rats treated with VOO. We used a dosage of VOO of 100 µL/day or 200 µL/day. As the mean weight of the rats was 320 g, we can extrapolate that if administered in a 60-kg human, this dosage would be 5.8 mL or 11.6 mL of VOO/day, which may approximate the actual intake in the Spanish diet. In a study in humans by Roncero et al., administration of VOO was 18.32 g/day, which is slightly higher than ours (18.32 g vs. 11.6 mL) [21]. The authors of the study found a significant increase in volumetric BMD. Otrowska et al. administered a very high dose of VOO to their pigs (19% (w/w) compared to the 100 µL/320 g given to our rats) [23]. The authors observed a substantial increase in BMD. Saleh and Saleh administered 1 mL/100 g of body weight to ovariectomized rats [24]. Extrapolating these dose levels to our study would have required doses of 3 mL/300 g, though the dose we used was 100 µL to 200 µL of VOO/day. Saleh and Saleh also observed a marked improvement in BMD in the group treated with VOO.

One interesting aspect of our results is the increase observed in D2D and D3D in the OVX rats treated with 200 µL of VOO. The values for the OVX rats treated with VOO increased with respect to untreated rats, although this measure did not reach the levels of the SHAM group. This increase indicates that the composition of bone in rats treated with 200 µL of VOO is more complex, more irregular, and, therefore, more similar to healthy bone.

Another interesting finding of our study is the increase in femur elasticity (Young’s modulus) along the x-axis in OVX animals who received doses of 100 µL or 200 µL of VOO. In rats given 100 µL, bone elasticity returned to the value of the SHAM group. In addition, the group of rats treated with 200 µL of VOO showed a recovery in femur elasticity along the z-axis, although these levels did not reach those recorded for the SHAM group. To our knowledge, the literature contains no previous studies that have found an effect of olive oil on biomechanical parameters.

With respect to biochemical markers of bone turnover, ovariectomy produced, as expected, an increase in BGP, denoting an increase in bone remodeling. We expected also an increase in TRAP levels in OVX rats, but we found a decrease in this bone marker that we cannot explain. The ingest of VOO did not produce any effect on variations in bone remodeling. This can explain the lack of variations found in BMD in treated OVX rats.

## 5. Conclusions

In this study, we found that OVX rats treated with a dose of VOO that resembles levels of VOO consumed in a normal human diet in Spain display no improvement in FBDM or LBMD but do evidence improved bone quality caused by a recovery of bone elasticity and fractal dimension (complexity of bone), returning to levels similar to those of healthy bone. Future experiments testing different polyphenols of VOO in these same conditions would be interesting. Our results therefore support those of previous works indicating that that dietary consumption of olive oil may improve some bone characteristics.

## References

[1] NIH (2001). Consensus Development Panel on Osteoporosis Prevention, Diagnosis, and Therapy. Osteoporosis prevention, diagnosis, and therapy. JAMA.

[2] Bonjour J.P. (2011). Protein intake and bone health. Int. J. Vitam. Nutr. Res..

[3] Pedrera-Zamorano J.D., Calderón-García J.F., Roncero-Martín R., Mañas-Núñez P., Morán J.M., Lavado-García J.M. (2012). The protective efecto of calcium on bone mass in postmenopausal women with high selenium intake. J. Nutr. Health Aging.

[4] Díaz Curiel M., Gil A., Mataix J. (2004). Nutrición y salud ósea.

[5] Keiler A.M., Zierau O., Bernhardt R., Scharnweber D., Lemonakis N., Termetzi A., Skaltsounis L., Vollmer G., Halabalaki M. (2014). Impact of a functionalized olive oil extract on the uterus and the bone in a model of postmenopausal osteoporosis. Eur. J. Nutr..

[6] Pelucci C., Bosetti C., Negri E., Lipworth L., La Vecchia C. (2010). Olive oil and cáncer risk: An update of epidemiological findings through. Curr. Pharm. Des..

[7] Kontigianni M.D., Melistas L., Yannakoulia M., Malagaris I., Panagiotakos D.B., Yiannakouris N. (2009). Association between dietary patterns and indices of bone mass in a sample of Mediterranean women. Nutrition.

[8] Rivas A., Romero A., Mariscal-Arcas M., Monteagudo C., Feriche B., Lorenzo M.L., Olea F. (2013). Mediterranean diet and bone mineral density in two age groups of women. Int. J. Food. Sci. Nutr..

[9] Savanelli M.C., Barrea L., Macchia P.E., Savastano S., Falco A., Renzullo A., Scarano E., Nettore I.C., Colao A., Di Somma C. (2017). Preliminary results demonstrating the impact of Mediterranean diet on bone health. J. Transl. Med..

[10] Silva T.D.R., Martins C.C., Ferreira L.L., Spritzer P.M. (2019). Mediterranean diet is associated with bone mineral density and muscle mass in postmenopausal women. Climateric.

[11] Garcia-Martinez O., Rivas A., Ramos-Torrecilla J., De Luna-Bertos E., Ruiz C. (2014). The effect of olive oil on osteoporosis prevention. Int. J. Food Sci. Nutr..

[12] Servili M., Esposto S., Fabiani R., Urbani S., Taticchi A., Mariucci F., Selvaggini R., Montedoro G.F. (2009). Phenolic compounds in olive oil: Antioxidant, health and organoleptic activities according to their chemical structure. Inflammopharmacology.

[13] Kanakis P., Termentzi A., Michel T., Gikas E., Halabaki M., Skaltsounis A.L. (2013). From olive drupes to olive oil. An HPLC-orbitrap-based qualitativeand quantitative exploration of olve key metabolites. Planta Med..

[14] Kim T.H., Jung J.W., Ha B.G., Hong J.M., Park E.K., Kim H.J., Kim S.Y. (2011). The effects of luteolin on osteoclast differentiation function in vitro and ovariectomy-inducedbone loss. J. Nutr. Biochem..

[15] Tagliaferri C., Davicco M.J., Lebecque P., Georgé S., Amiot M.J., Mercier S., Dhaussy A., Huertas A., Walrand S., Wittrant Y. (2014). Olive oil and vitamin D synergistically prevent bone loss in mice. PLoS ONE.

[16] Borchers R.E., Gibson L.J., Burchardt H., Hayes W.C. (1995). Effects of selected thermal variables on the mechanical properties of trabecular bone. Biomaterials.

[17] Baum T., Carballido-Gamio J., Huber M.B., Müller D., Monetti R., Räth C., Eckstein F., Lochmüller E.M., Majumdar S., Rummeny E.J. (2010). Automated 3D trabecular bone structure analysis of the proximal femur—Prediction of biomechanical strength by CT and DXA. Osteoporos. Int..

[18] Alberich-Bayarri A., Martí-Bonmatí L., Sanz-Requena R., Sánchez-González J., Briz V.H., García-Martí G., Pérez M.Á. (2014). Reproducibility and accuracy in the morphometric and mechanical quantification of trabecular bone from 3 Tesla Magnetic resonance images. Radiologia.

[19] Alberich-Bayarri A., Marti-Bonmati L., Pérez M.A., Sanz-Requena R., Lerma-Garrido J.J., García-Martí G., Moratal D. (2010). Assesment of 2D and 3D fractal dimension measurements of trabecular bone from high-spatial resolution magnetic resonance images in 3T. Med. Phys..

[20] Alberich-Bayarri A., Moratal D., Ivirico J.L.E., Hernández J.C.R., Vallés-Lluch A., Martí-Bonmatí L., Estellés J.M., Mano J.F., Pradas M.M., Ribelles J.L.G. (2009). MIcrocomputed tomography and microfinite element modeling for evaluating polymer scaffolds architecture and their mechanical properties. J. Biomed. Mater. Res. B Appl. Biomater..

[21] Roncero-Martín R., Aliaga Vera I., Moreno-Corral L.J., Moran J.M., Lavado-Garcia J.M., Pedrera-Zamorano J.D., Pedrera-Canal M. (2018). Olive oil consumption and bone microarchitecture in Spanish Women. Nutrients.

[22] Rivas A., Romero A., Mariscal M., Monteagudo C., Hernandez J., Olea-Serrano F. (2009). Validation of questionnaries for the study of food habits and bone mass. Nutr. Hosp..

[23] Ostrowska E., Gabler N.K., Ridley D., Suster D., Eagling D.R., Dunshea F.R. (2006). Extra-virgin and refined olive oil decrease plasma triglyceride, moderately affect lipoprotein oxidation susceptibility and increase bone density in growing pigs. J. Sci. Food Agric..

[24] Saleh N.K., Saleh H.A. (2011). Olive oil effectively mitigates ovariectomy-induced osteoporosis in rats. BMC Complement. Altern. Med..

[25] Santiago-Mora R., Casado-Díaz A., De Castro M.D., Quesada-Gómez J.M. (2011). Oleuropein enhances osteoblastogenesis and inhibits adipogenesis: The effect on differentiation in stem cells derived from bone marrow. Osteoporosis. Int..

[26] Puel C., Quintin A., Agalias A., Mathey J., Obled C., Mazur A., Davicco M.J., Lebecque P., Skaltsounis A.L., Coxam V. (2004). Olive oil and its main phenolic micronutrient (oleuropein) prevent inflammation-induced bone loss in the ovariectomosed rat. Br. J. Ntr..

[27] Hagiwara K., Goto T., Araki M., Miyazaki H., Hagiwara H. (2011). Olive polyphenol hydroxytyrosol prevents bone loss. Eur. J. Pharmacol..

[28] Melguizo-Rodriguez L., Manzano Moreno F.J., De Luna-Bertos E., Rivas A., Ramos-Torrecillas J., Ruiz C., García-Martínez O. (2018). Effect of olive phenolic compounds on osteoblast differentiation. Eur. J. Clin. Investig..

